# Dramatic response to immunochemotherapy followed by salvage surgery in an elderly lung cancer patient

**DOI:** 10.1111/1759-7714.14293

**Published:** 2021-12-20

**Authors:** Yoshio Okano, Michihiro Kunishige, Yoshihiro Kondo, Naoki Kadota, Atsushi Morishita, Keishi Naruse, Hisanori Machida, Nobuo Hatakeyama, Hiroyuki Hino, Tsutomu Shinohara, Shoji Sakiyama, Eiji Takeuchi

**Affiliations:** ^1^ Department of Respiratory Medicine National Hospital Organization Kochi Hospital Kochi City Japan; ^2^ Department of Thoracic Surgery National Hospital Organization Kochi Hospital Kochi City Japan; ^3^ Department of Pathology National Hospital Organization Kochi Hospital Kochi City Japan; ^4^ Department of Community Medicine for Respirology Graduate School of Biomedical Sciences, Tokushima University Tokushima Japan; ^5^ Department of Clinical Investigation National Hospital Organization Kochi Hospital Kochi City Japan

**Keywords:** advanced squamous cell carcinoma of the lung, immune checkpoint inhibitor, induction immunochemotherapy, pathological complete response

## Abstract

Immune checkpoint inhibitors (ICIs) have caused a paradigm shift in the treatment of lung cancer. Here, we encountered a case of inoperable locally advanced squamous cell carcinoma of the lung that became operable with pembrolizumab‐based immunochemotherapy and achieved a pathological complete response. An 82‐year‐old man suspected of having lung cancer was referred to our hospital. The patient was clinically diagnosed with left upper lobe squamous cell carcinoma cT2aN3M0 c‐stage IIIC. Immunostaining revealed the expression of programmed death‐ligand 1 in 60% of tumor cells. The cancer cells disappeared after two cycles of chemotherapy with carboplatin and nanoparticle albumin‐bound paclitaxel plus pembrolizumab. As the abnormal accumulation of ^18^F‐fluorodeoxyglucose (FDG) on FDG‐positron emission tomography/computed tomography before chemotherapy almost disappeared after pembrolizumab‐based immunochemotherapy, left upper lobectomy and lymph node dissection were performed. No cancer cells were pathologically detected from the resected tissue. Therefore, ICIs combined with chemotherapy may enable inoperable advanced lung cancer patients to undergo surgery and achieve a complete response.

## Graphical abstract

Immune checkpoints inhibitors (ICIs) combined with chemotherapy may enable inoperable advanced lung cancer patients to undergo surgery and achieve a complete response.

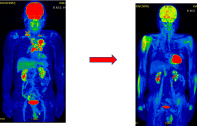

## INTRODUCTION

Immune checkpoint inhibitors (ICIs) have caused a paradigm shift in the treatment of lung cancer. The safety and efficacy of neoadjuvant ICIs alone and in combination with chemotherapy are currently being investigated.^1^, ^2^, ^3^, ^4^, ^5^, ^6^ In addition, cases of inoperable advanced non‐small cell lung cancer (NSCLC) that became operable with ICIs or ICIs in combination with chemotherapy and achieved a complete response (CR) have previously been reported.^7^, ^8^, ^9^, ^10^, ^11^ However, further research is needed on the combination of ICIs and chemotherapy as induction therapy for advanced NSCLC because their efficacy and safety have not yet been established.

Here, we encountered a case of inoperable locally advanced squamous cell carcinoma of the lung that became operable with pembrolizumab‐based immunochemotherapy and achieved pathological CR. We herein present and describe this case.

## CASE REPORT

**FIGURE 1:**
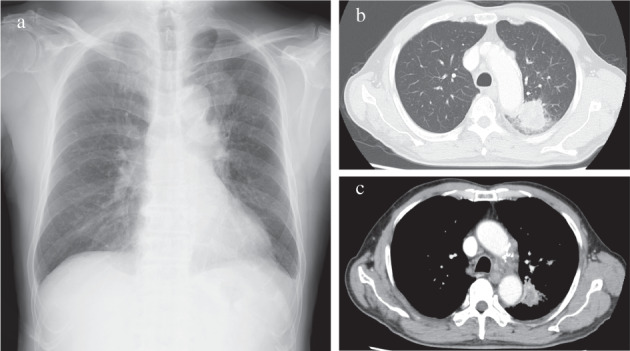
(a) Chest X‐ray before treatment. (b, c) Chest computed tomography (CT) before treatment. A 35 mm tumor was detected in the left upper lobe

**FIGURE 2:**
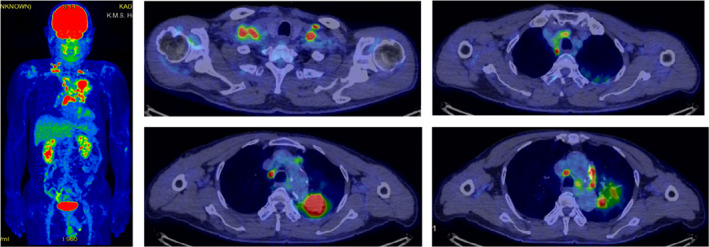
^18^F‐fluorodeoxyglucose (FDG)‐positron emission tomography/computed tomography (PET/CT) at pretreatment. An accumulation of FDG was observed in the left upper lobe (SUVmax: 15.1) and multiple lymph nodes. SUVmax: maximum standardized uptake value

**FIGURE 3:**
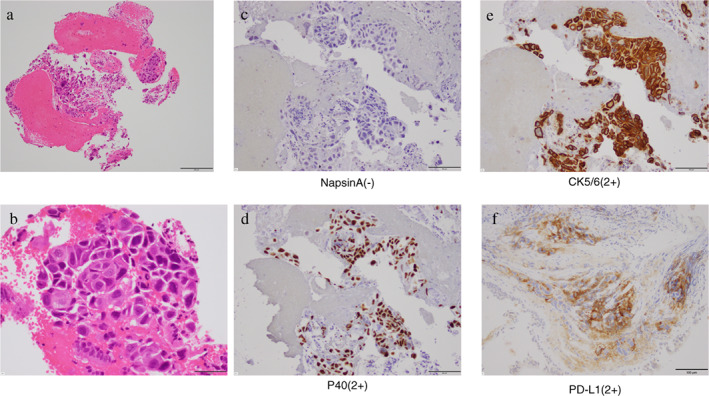
Histopathological examination using hematoxylin–eosin staining (a, b) (bar = 200 μm, a) (Bar = 50 μm, b) and immunohistochemical staining (c–f). Napsin A (bar = 100 μm, c) was negative, while P40 (bar = 100 μm, d) and CK5/6 (bar = 100 μm, e) were both strongly positive. Squamous cell carcinoma was diagnosed based on these findings. Immunostaining revealed that PD‐L1 was expressed on 60% of tumor cells (22C3 clones) (bar = 100 μm, f)

**FIGURE 4:**
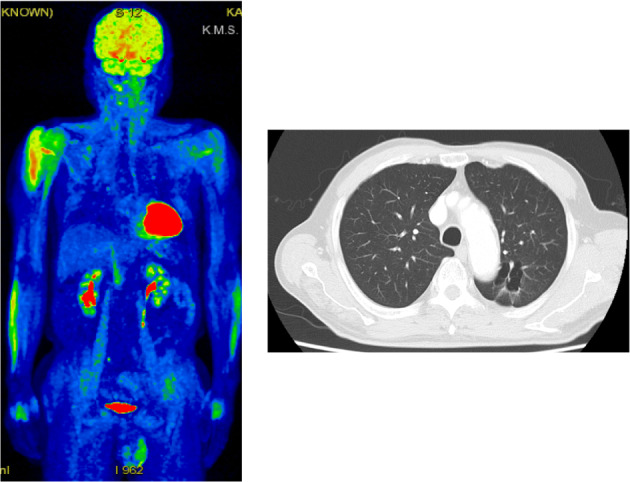
^18^F‐fluorodeoxyglucose (FDG)‐positron emission tomography/computed tomography (PET/CT) after pembrolizumab‐based immunochemotherapy. The accumulation of FDG disappeared

An 82‐year‐old man suspected of having lung cancer was referred to our hospital. He had no chief complaint with a medical history of gastric cancer 2 years previously, diabetes, hypertension, and dyslipidemia. He had smoked two packets of cigarettes per day for 50 years. His Eastern Cooperative Oncology Group performance status was 0. Blood tests showed elevated levels of carcinoembryonic antigen (5.9 ng/ml) and cytokeratin 19 fragments (36 ng/ml). The neutrophil‐to‐lymphocyte ratio was 2.3. A tumor was detected in the left upper lobe on chest X‐ray (Figure 1a) and contrast‐enhanced computed tomography (CT) (Figure 1b,c). ^18^F‐fluorodeoxyglucose (FDG)‐positron emission tomography/CT (PET/CT) showed a very high uptake of FDG in the left upper lobe (maximum standardized uptake value [SUVmax] of 15.1) and multiple lymph nodes (Figure 2). Transbronchial biopsy was performed and the sample obtained was histopathologically diagnosed as squamous cell carcinoma (Figure 3). Immunostaining revealed that programmed death‐ligand 1 (PD‐L1) was expressed on 60% of tumor cells (22C3 clones) (Figure 3). An Oncomine Dx target test was negative. No other distant metastases were detected, and the patient was clinically diagnosed with left upper lobe squamous cell carcinoma c‐T2aN3M0 c‐stage IIIC. Daily carboplatin combined with radiation therapy was considered, but abandoned due to concerns regarding the size of the irradiated area and the age of the patient. The patient looked younger than his calendar age, and we decided that pembrolizumab‐based immunochemotherapy was feasible. Therefore, treatment with carboplatin (area under the plasma concentration‐time curve 6, day 1) and nanoparticle albumin‐bound paclitaxel (nab‐PTX) (100 mg/m^2^, days 1, 8, and 15) plus pembrolizumab (200 mg/bodyweight, day 1) was initiated. Carboplatin and nab‐PTX plus pembrolizumab were administered in two cycles. There were no adverse events of note. Contrast‐enhanced CT showed that cancer cells had disappeared. The abnormal accumulation of FDG on FDG‐PET/CT before chemotherapy had almost disappeared after immunochemotherapy (Figure 4). However, there was a possibility that microscopic cancer cells remained in the lungs and lymph nodes. After providing a sufficient explanation and obtaining consent from the patient himself and his family, left upper lobectomy and lymph node dissection were performed. No cancer cells were detected pathologically from the resected tissue. We considered the need for adjuvant chemotherapy. However, postoperative immunochemotherapy or cisplatin and vinorelbine were not performed at the patient's request. In the 11 months since surgery and therapy, there have been no signs of recurrence and the condition of the patient is good.

## DISCUSSION

We encountered a case of inoperable locally advanced squamous cell carcinoma of the lung that became operable with pembrolizumab‐based immunochemotherapy and achieved pathological CR. To the best of our knowledge, case reports of inoperable advanced squamous cell carcinoma that became operable with ICIs and chemotherapy, leading to CR, are extremely rare.

Pembrolizumab is a humanized monoclonal antibody against programmed death 1 with antitumor activity for NSCLC.^12^, ^13^ In the KEYNOTE‐024 trial, pembrolizumab monotherapy achieved significantly longer progression‐free (PFS) and overall survival (OS) than platinum‐based chemotherapy for PD‐L1‐positive NSCLC.^14^ In the KEYNOTE‐189 trial, the addition of pembrolizumab to standard chemotherapy resulted in significantly longer OS and PFS than chemotherapy.^15^ In the KEYNOTE‐407 trial, the addition of pembrolizumab to carboplatin and either paclitaxel or nab‐PTX resulted in significantly longer OS and PFS than chemotherapy alone for squamous cell carcinoma.^16^ The safety and efficacy of neoadjuvant ICIs alone or in combination with chemotherapy are currently being investigated.^1^, ^2^, ^3^, ^4^, ^5^, ^6^

ICI monotherapy or ICIs in combination with chemotherapy have previously been applied to the treatment of patients with inoperable advanced NSCLC, and after the confirmation of tumor reductions, surgery was performed and no tumor cells were detected.^7^, ^8^, ^9^, ^10^, ^11^ The present case had more extensive disease and was older than these cases. Although surgery after immunochemotherapy is controversial, it is appropriate because the affected area may be excised and subjected to a histopathological examination. One limitation is that although the abnormal accumulation of FDG in the bilateral supraclavicular fossa lymph nodes was confirmed on FDG‐PET/CT before treatment, it was not possible to histopathologically exclude residual tumor cells in the same area because resection was not performed. Therefore, the possibility of residual tumor cells needs to be considered. Furthermore, since postoperative immunochemotherapy or chemotherapy was not performed at the patient's request, a strict follow‐up for metastasis and recurrence is required.

In conclusion, we encountered a case of inoperable locally advanced squamous cell carcinoma of the lung that became operable with pembrolizumab‐based immunochemotherapy and achieved pathological CR. ICIs combined with chemotherapy may enable inoperable advanced lung cancer patients to undergo surgery or achieve CR.

## CONFLICT OF INTEREST

The authors declare no conflicts of interest.
